# Is hepatotropic contrast enhanced MR a more effective method in differential diagnosis of hemangioma than multi-phase CT and unenhanced MR?

**DOI:** 10.1186/1471-230X-11-43

**Published:** 2011-04-19

**Authors:** Edyta Szurowska, Tomasz Nowicki, Ewa Izycka-Swieszewska, Joanna Wypych, Anna Drobinska-Jurowiecka, Karolina Markiet, Arkadiusz Szarmach, Michal Studniarek

**Affiliations:** 1 Department of Radiology, Medical University of Gdansk, Debinki 7, 80-211 Gdansk, Poland; 2 Department of Pathology, Medical University of Gdansk, Debinki 7, 80-211 Gdansk, Poland; 3 Department of Gastroenterology and Hepatology, Medical University of Gdansk, Debinki 7, 80-211 Gdansk, Poland; 4 Department of Neonatal, Gynecological and Urological Radiology, Medical University of Gdansk, Debinki 7, 80-211 Gdansk, Poland

## Abstract

**Background:**

Cavernous hemangiomas are the most frequent neoplasms of the liver and in routine clinical practice they often need to be differentiated from malignant tumors and other benign focal lesions. The purpose of this study is to evaluate whether diagnostic accuracy of magnetic resonance imaging (MRI) of hepatic hemangiomas, showing atypical pattern on US, improves with the use of Gd-BOPTA in comparison with contrast-enhanced multi-phase computed tomography (CT).

**Methods:**

178 consecutive patients with ambiguous hepatic masses showing atypical hyperechoic pattern on grey-scale US, underwent unenhanced and contrast-enhanced multi-phase multi-detector CT and MR (1.5T) with the use of liver-specific contrast medium gadobenate dimeglumine (Gd-BOPTA). After intravenous contrast administration arterial (HAP), venous-portal (PVP), equilibrium phases (EP) both in CT and MR and additionally hepatobiliary phase (HBP) in MR were obtained. 398 lesions have been detected including 99 hemangiomas and 299 other lesions.

**Results:**

In non-enhanced MDCT examination detection of hemangiomas was characterized by sensitivity of 76%, specificity of 90%, PPV of 71%, NPV of 92% and accuracy of 86%.

Non-enhanced MR examination showed sensitivity of 98%, specificity of 99%, PPV of 99%, NPV of 99% and accuracy of 99%.

After intravenous administration of contrast medium in MR the mentioned above parameters did not increase significantly.

**Conclusion:**

Gd-BOPTA-enhanced MR in comparison with unenhanced MRI does not improve diagnostic accuracy in discriminating hemangiomas that show non-specific appearance in ultrasound examination. Unenhanced MR as a method of choice should directly follow US in course of diagnostic algorithm in differentiation of hemangiomas from other liver tumors.

## Background

Cavernous hemangiomas are the most frequent benign neoplasms of the liver, found in autopsy examinations within 0.4% to 20% of population [1,2]. Such frequent occurrence of hepatic hemangiomas (HH) rises a need for differential diagnosis between those benign lesions and malignant liver tumors, especially metastases. Many HH show characteristic image in US examination. However, in some cases hemangiomas show a non-specific appearance in baseline B-mode US [3,4] and contrast-enhanced ultrasound (US), computed tomography (CT) or magnetic resonance (MR) examinations are applied for further evaluation of observed focal liver lesions. An atypical hemangioma in US usually shows echogenic border, internal echogenic pattern at least partially hypoechogenic, totally hypoechogenic or heterogenic pattern or may contain calcifications or appear as large lesion [5].

Great technological development concerning both multi-detector computer tomography and hepatocyte-specific contrast agents in magnetic resonance imaging (MRI) has been observed in recent years.

Computed tomography is a well established procedure of imaging of hepatic hemangiomas. Most hemangiomas show typical enhancement pattern: nodular or globular peripheral enhancement in arterial phase with a progressive fill-in in portal venous and equilibrium phases.

In particular cases the use of magnetic resonance imaging leads to specification of diagnosis based on US and CT findings.

Many studies have been dedicated to differential diagnosis of hemangiomas from malignant tumors [6-8]. Previous studies showed that the use of hepatocyte-specific contrast agents improves the accuracy and confidence of diagnosis of focal liver lesions, however, to our best knowledge none compared gadobenate dimeglumine (Gd-BOPTA) enhanced MRI with unenhanced MR and CT in differentiation of hemangiomas showing atypical US appearance from other focal liver lesions [9,10]. Any imaging technique used for verification of atypical hemangiomas should be validated.

The purpose of this study is to evaluate whether assessment by means of MRI of hepatic hemangiomas, showing atypical appearance on gray-scale US, improves with the use of Gd-BOBTA in comparison with contrast-enhanced multiphase CT.

## Methods

178 consecutive patients with non-specific hepatic foci observed in US were included in this prospective study and spiral CT and MR examinations were performed in those patients within the period not exceeding 3 weeks.

**Figure 1:**
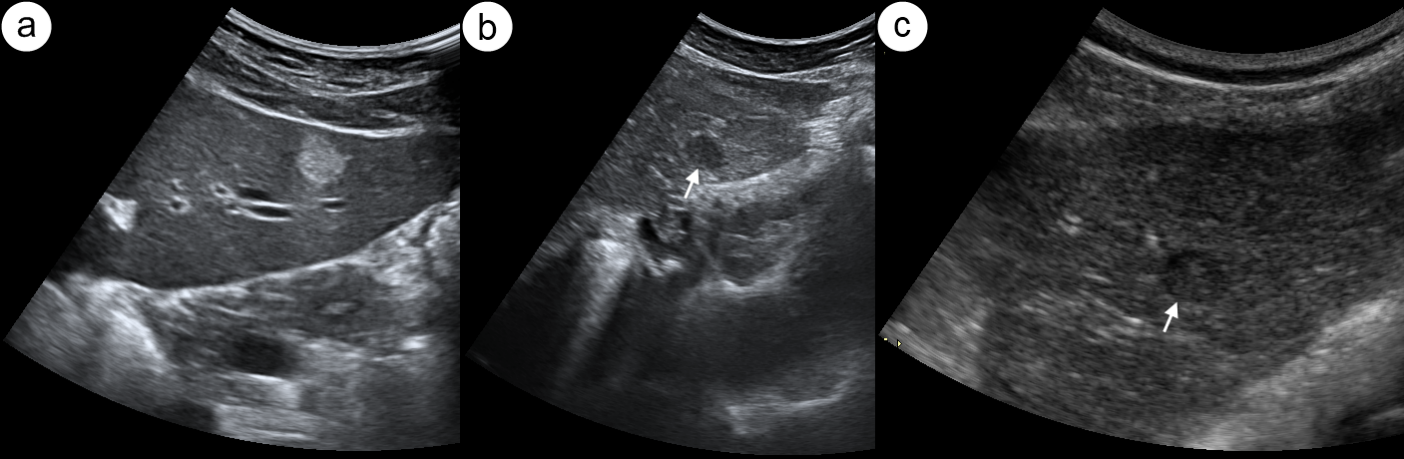
**Hepatic hemangioma in US**. Transverse sonogram of left lobe of liver presents typical hyperechogenic hemangioma (a). Figure 1b and c show two different hypoechogenic liver lesions suspected to be atypical hemangioma, subsequently confirmed in CT.

Inclusion criteria were as follows: presence of focal liver lesion that in grey-scale US did not present typical features of a simple cyst (anechoic focus with posterior acoustic enhancement) or a typical hemangioma (hyperechoic oval pattern with homogeneous or slightly inhomogeneous echotexture, well-defined margins and posterior wall shadowing, figure 1a). The atypical hemangiomas included in the study were characterized on US by echogenic border, internal echogenic pattern at least partially hypoechogenic or totally hypoechogenic pattern (figure 1b, c).

Exclusion criteria included any contraindications to CT or MR examination, amongst them contraindications to iodine contrast agents or Gd-BOPTA administration and lack of patient's consent.

The standard of reference was the histopathological examination or clinical and imaging follow-up for the duration of 18 months in case of HH and FNH, 12 months in case of inflammatory lesions and 3 to 24 months in patients suffering from liver metastases treated with chemotherapy.

MR study was performed with 1.5 T MR system using phased-array flex coil. The protocol included: T1-weighted SE sequence (TR/TE ms - 303/12, scan time - 17sec) in axial and coronal plane with and without contrast enhancement in equilibrium and hepatobiliary phase, T2-weighted Express sequence (18000/92, scan time - 17sec) in axial plane, T2-weighted FSE (6500/116.8, scan time - 20sec) in coronal plane, performed also with fat saturation and T1-weighted out of phase sequence (150/2.24, flip angle of 90°, scan time - 12sec) without and after contrast administration in hepatic arterial dominant phase (HAP), portal venous dominant phase (PVP), equilibrium phase (EP) and hepatobiliary phase (HBP). Slice thickness was 5mm, intersection gap - 0.5, matrix 256 × 256. Dynamic MRI was obtained immediately after a bolus injection of Gd-BOPTA (dosage of 0.1 mmol per kilogram of body weight), followed by saline solution flush of 25mL through a 20G venous catheter positioned in the antecubital vein. Similarly to the multiphase CT study HAP, PVP, and EP were performed after respectively 25, 60 and 180sec. HBP was acquired 60min after contrast agent administration. The phase-encoding direction was anterior-posterior for all sequences. All images were acquired during breath-hold.

Multi-phase multi-detector (6 and 32-row) CT examination of the liver has been conducted first unenhanced, then after application of a contrast agent in HAP, PVP and EP. 100ml of iodinated contrast agent at a rate of 4mL/sec through an 20G venous catheter positioned in an antecubital vein using a power injector was administrated. The CT section thickness was 2.5mm, images interval - 2.5mm and pitch - 1.0.

Spiral CT and MRI were then interpreted by three independent radiologists with at least 10-years (ES, MS) and 5-years (AS) experience in abdominal imaging, who had no previous knowledge of patients' medical history. The interval between CT and MR examinations was from 3 to 5 weeks. Final assessment was based on the observers' consensus in case of disagreement.

Radiological parameters assessed in CT and MR analysis were: size of detectable lesions, their density, signal intensity and type of enhancement.

**Figure 2:**
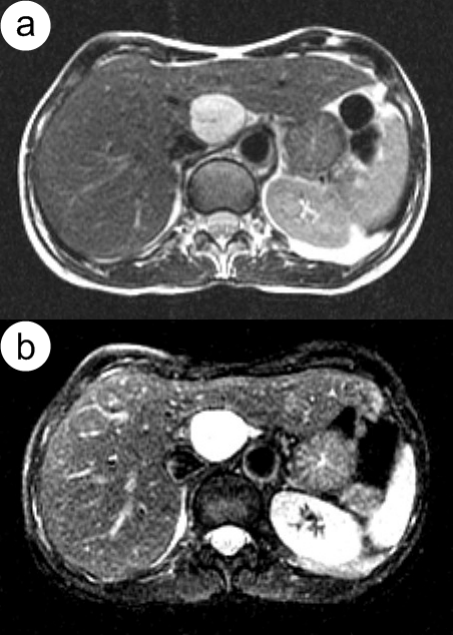
**Hepatic hemangioma**. Moderately (a) and heavily (b) T2-weighted MR images show typical bright lesions.

Signal intensity of each lesion in relation to adjacent liver parenchyma in T2-weighted TSE images was assessed. A 4-point scale has been used: 1-hypointense, 2-isointense, 3-hyperintense and 4-bright lesion (marked hyperintensity, figure 2).

Type of enhancement in hepatic arterial phase was qualified as one of the following: intensive homogenous, heterogenous, nodular or globular peripheral enhancement, partial or complete ring shape and no enhancement.

In portal venous and equilibrium phase one of the following enhancement type was assigned to each visible lesion: homogenous, heterogenous, progressive fill-in, partial or complete ring shape, no enhancement and enhancement similar to liver.

Each focus was assessed by independent observers in MDCT, unenhanced MRI and Gd-BOPTA-enhanced MRI and classified into one of the groups: hemangioma or non-hemangioma.

In unenhanced MRI hemangioma was defined as a lesion presenting low signal intensity in T1-weighted images, while in T2- and heavily T2-weighted images remaining highly homogenous, clearly demarcated from the adjacent liver parenchyma, showing very high signal intensity, similar to that of cerebrospinal fluid, due to the long T2 relaxation time of its blood-filled vascular channels. This feature has been described as the bulb-light sign.

In dynamic contrast-enhanced CT and MRI hemangiomas were defined when showing peripheral globular enhancement and a centripetal fill-in pattern with the attenuation of enhancing areas corresponding to that of the aorta and blood pool.

Hemangiomas were diagnosed in Gd-BOPTA-enhanced MRI when showing typical features of hemangioma in unenhanced-MRI and/or in dynamic contrast-enhanced MR examination.

These criteria have been previously applied by many authors [11-14].

Foci were evaluated as of a different type than hemangiomas when not presenting any of the above mentioned features.

All lesions were divided into two groups: the first group (HH group) consisted only of liver hemangiomas, the second group (nonHH group) included focal liver lesions other than hemangiomas.

All lesions were also classified as small or large according to their medians by the study coordinator (ES).

The interobserver agreement was measured with Cohen's kappa coefficient. Statistical analysis of size of detectable lesions, their density, signal intensity and type of enhancement of hemangiomas and other lesions in unenhanced, gadolinium-enhanced MR and CT studies has been performed with Statistica 8 software (StatSoft Inc., Tulsa, OK, USA) and McNemar's test with Bonferroni correction for multiply comparison. *P *values less than 0.05 were considered as statistical significant.

The research was approved by Independent Bioethic Committee for Scientific Research of Medical University of Gdansk (NKEBN/649/2001-2002). All patients gave their written consent to participate.

The study has been partially financed from the research grant ST-82 given by authorities of the Medical University of Gdansk to MS.

## Results

178 patients were qualified for the study, amongst this group 161 underwent further analysis (95 women and 66 men, age 18-79 years).

In case of 17 patients we failed to establish the final diagnosis - 2 of the patients passed away, 15 did not show for the follow-up.

High interobserver agreement (kappa values of 0.80-0.99) confirmed the methodology as reliable and reproducible.

In 100 patients the final diagnosis was based upon histopathological examination results (66 cases by the means of surgery and 34 cases with biopsy) which disclosed: 41 cases of HCC, 21 FNHs, 28 cases of liver metastases, 4 cavernous hemangiomas, 4 solitary adenomas and 2 cases of peripheral cholangiocarcinoma.

In the remaining 61 patients, the final diagnosis was based upon the clinical and diagnostic imaging follow-up. In this group we observed: 30 hepatic hemangiomas (confirmed in contrast-enhanced US examination and follow-up MR examination), 18 FNHs (confirmed in Gd-EOB-DTPA-enhanced MR), 13 cases of liver metastases (treated with chemotherapy and confirmed in follow-up CT examinations) and 2 inflammatory lesions (follow-up CT examinations showed no lesions).

The HH group consisted of 99 liver hemangiomas, recognized in 34 patients. 21 patients with HH had isolated liver lesions and multifocal HHs were recognized in 13 people.

The nonHH group included 127 patients with 299 focal liver lesions other than hemangioma: 144 metastases, 100 cases of hepatocellular carcinoma, 4 adenomas, 44 FNHs and 7 other lesions.

The diameter of foci ranged from 6mm to 125mm (median 21mm). According to the mean foci diameter, all lesions were divided into two groups: small lesions (<2cm) - 199 foci and large lesions (≥2cm) - 199 foci. In the HH-group, 40 foci of cavernous hemangiomas were large lesions and the rest of 59 tumors were small lesions.

Small hemangiomas more frequently (30/59) showed homogenous enhancement pattern in all three phases, while large hemangiomas (25/40) presented nodular or globular peripheral enhancement pattern in HAP and progressive fill-in in PVP and EP in dynamic MR study (table 1). The type of enhancement in consecutive phases was completely the same in both CT and MR studies.

**Table 1:** Relation between the enhancement pattern and detectable lesions in HAP and PVP in three groups: small, large and all lesions.

		HH			nonHH		
		
		small	large	all size	small	large	all size
HAP	homogenous	30	11	41	46	16	62
	
	heterogenous	1	2	3	1	19	20
	
	nodular peripheral	9	25	34	4	7	11
	
	ring shape	10	1	11	77	93	170
	
	no enhancement	9	1	10	12	24	36

PVP	homogenous	32	11	43	16	9	25
	
	heterogenous	1	1	2	2	10	12
	
	progressive fill-in	9	25	34	2	4	6
	
	ring shape	4	1	5	67	81	148
	
	similar to liver	13	2	15	53	55	108

**Figure 3:**
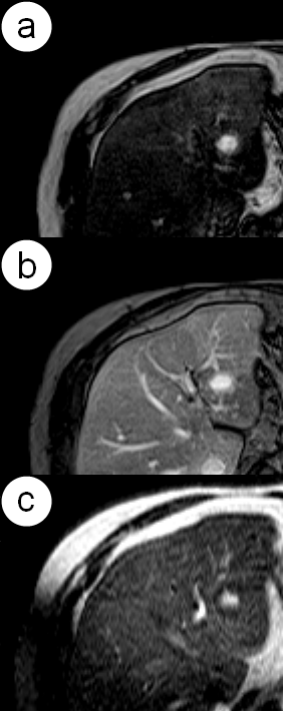
**Hepatic hemangioma**. MR study presents a homogeneous pattern of enhancement clearly visible in hepatic arterial (a) and portal venous (b) phases. T2-weighted MR image shows typical radiological finding of hemangioma - marked hyperintensity (c).

**Figure 4:**
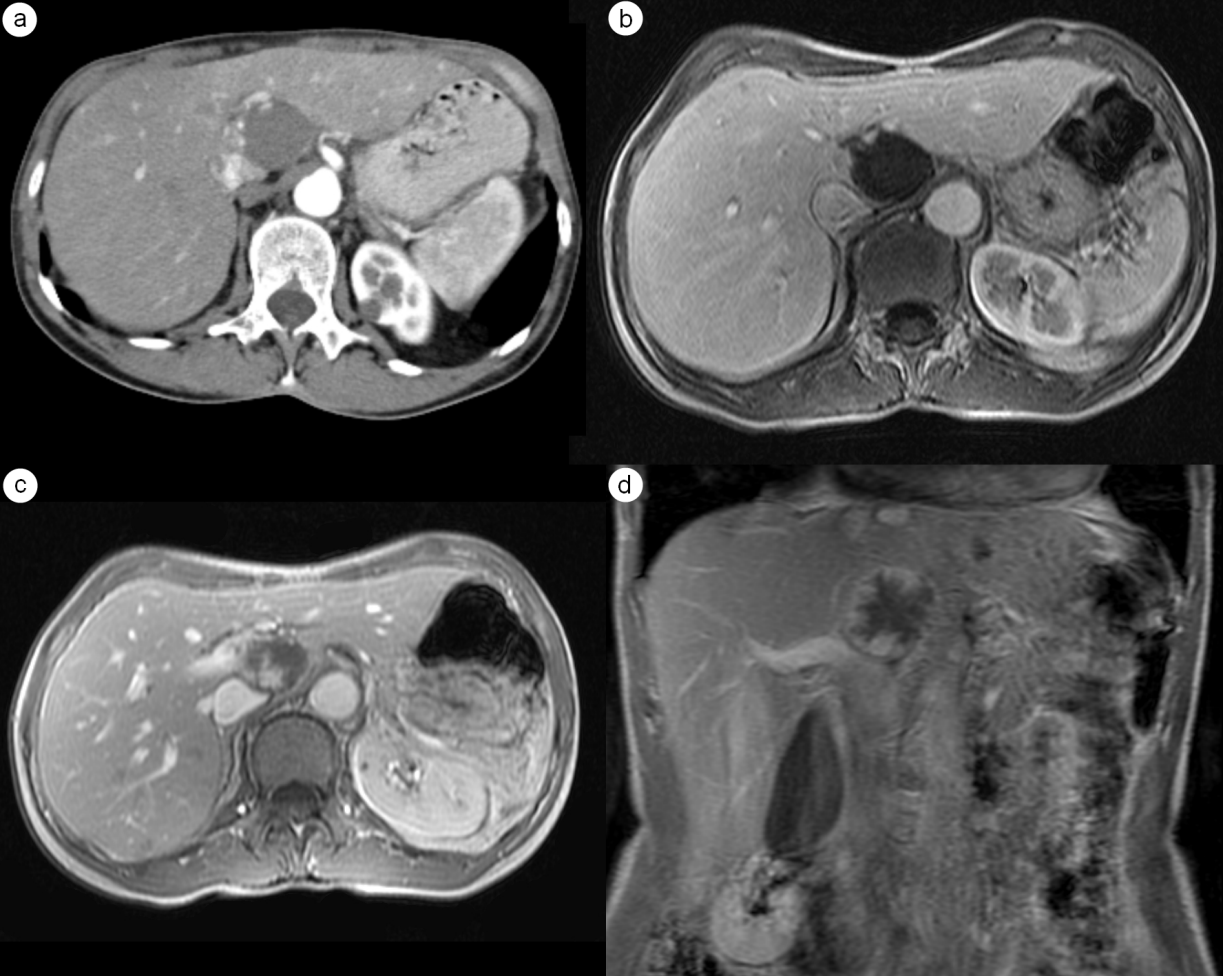
**The same case of the hepatic hemangioma as in figure 2**. HAP-CT image clearly visualizes globular type of enhancement (a). Globular pattern of enhancement is also visible in HAP - MR image (b). Progressive fill-in enhancement pattern can be observed in PVP (c) and EP (d) phases of MR study.

Gd-BOPTA - enhanced MR and multi-phase spiral CT evaluation of hepatic hemangiomas was based upon typical enhancement pattern in three phases (table 2):

- homogenous in HAP and PVP or EP (figure 3),

- nodular peripheral or globular in HAP with progressive fill-in enhancement in PVP and EP (figure 4).

**Table 2:** Relation between typical enhancement pattern for hemangioma in successive phases of dynamic CT/MR studies (homogeneous enhancement in HAP, PVP and EP or nodular peripheral/globular enhancement in HAP with progressive fill-in enhancement in PVP and EP) and detectable lesions in three groups: small, large and all lesions.

Groups and subgroups	Homogeneous enhancement in HAP, PVP and EP (number of foci)	Nodular peripheral/globular enhancement in HAP with progressive fill-in enhancement in PVP and EP (number of foci)
small HH-group	30	9

small nonHH-group	20	1

large HH-group	11	25

large nonHH-group	5	5

all size HH-group	41	34

all size nonHH-group	25	6

**Figure 5:**
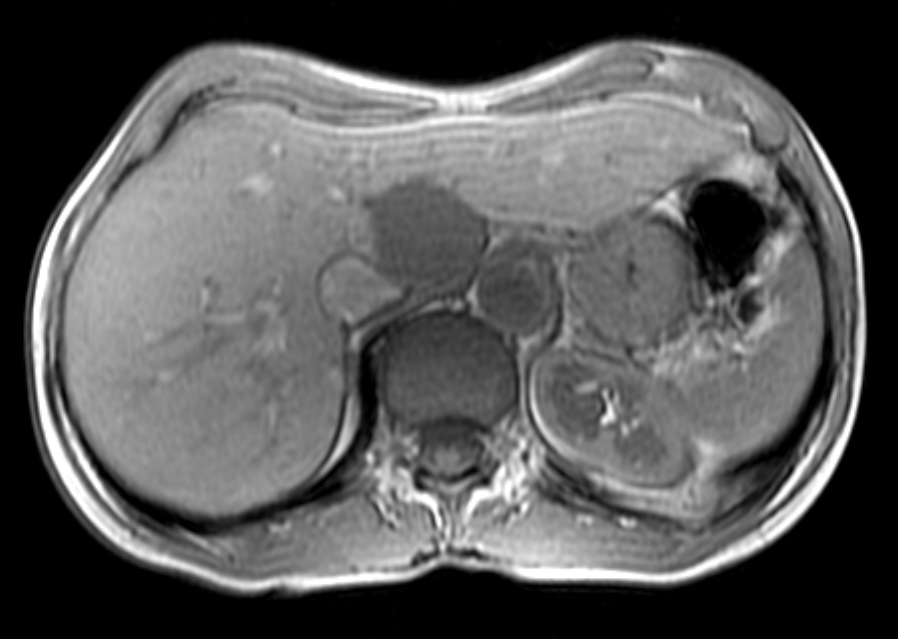
**The same case of the hepatic hemangioma as in figures 2 and 4 **. Hepatobiliary phase in MR presents weaker enhancement of this lesion in comparison to the adjacent liver parenchyma.

In the hepatobiliary phase of MR examination all hemangiomas showed a lower signal in comparison to the adjacent liver tissue (figure 5). 233 nonHH lesions were hypointense, 63 isointnese and 3 hyperintense.

Spiral CT correctly characterized 75 hemangiomas.

Signal intensity observed in moderately and heavily T2-weighted images in hemangiomas and other liver lesions is presented in table 3.

**Table 3:** Relation between signal intensity in moderately and heavily T2-weighted images and type of detectable lesions in three groups: small, large and all lesions.

	signal intensity in moderately and heavily T2-weighted images of all lesions			
	
	hypointense	isointense	hyperintense	markedly hyperintense
small HH	0	0	0	59

small nonHH	19	52	69	0

large HH	0	0	1	39

large nonHH	10	34	114	1

all size HH	0	0	1	98

all size nonHH	29	86	183	1

In unenhanced MR false-negative diagnosis was obtained in one case of hemangioma and one false-positive diagnosis of hemangioma was noted in case of cystic metastasis from ovarian carcinoma.

**Figure 6:**
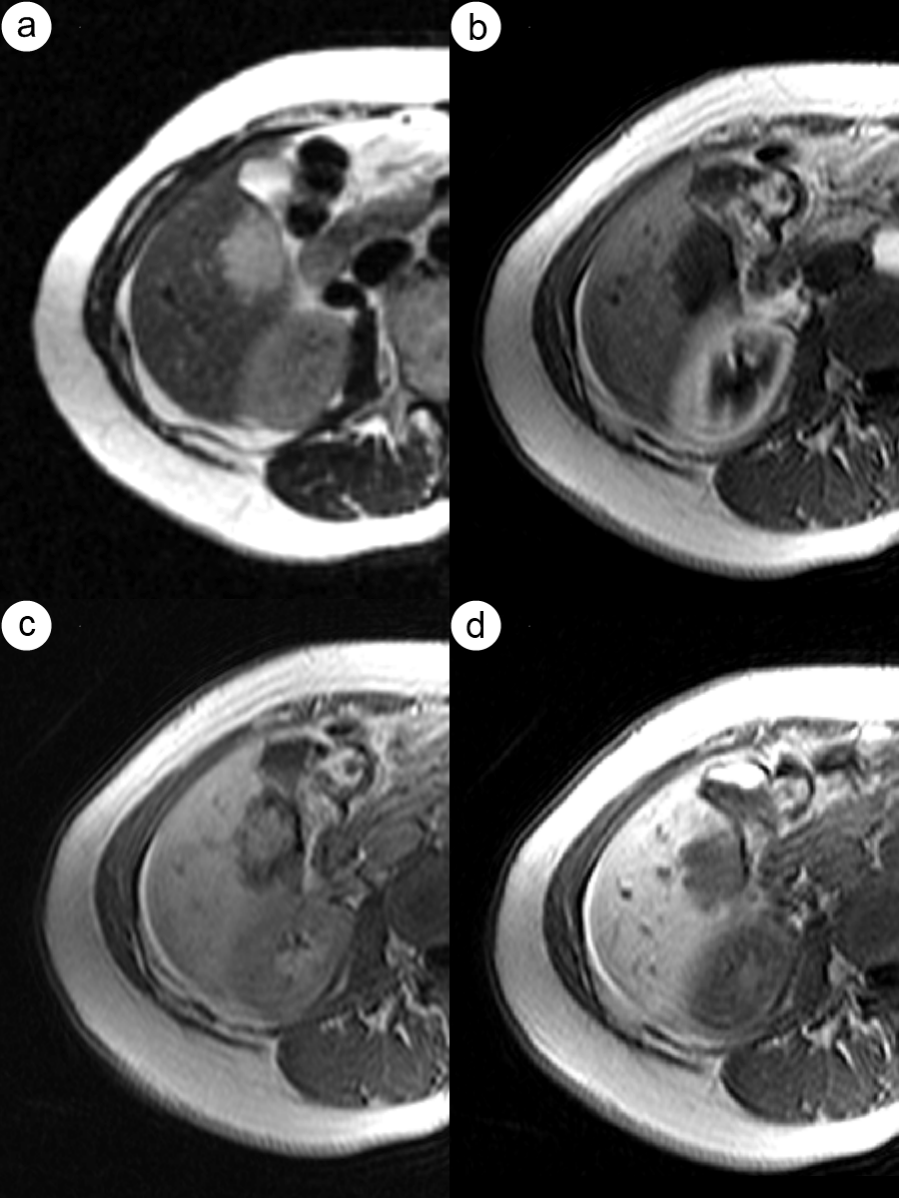
**Hepatic hemangioma with atypical enhancement pattern in dynamic MR study**. Moderately T2-weighted MR image with untypical weak intensity of the lesion (a). In HAP (b) enhancement of the hemangioma is not visible. Figure 6c shows central enhancement of the lesion in EP. The hemangioma presents weaker enhancement than the adjacent liver parenchyma in hepatobiliary phase (d).

Gd-BOPTA-enhanced MR study allowed to correctly diagnose cystic metastasis from ovarian carcinoma and therefore revealed 98 hemangiomas. Still one hemangioma was unrecognized (figure 6).

Unenhanced MR and Gd-BOPTA-enhanced MR show higher diagnostic accuracy in differentiation of hemangiomas than CT (*p *< 0.0001 for both modalities), also in case of small (*p *< 0.0001 for both modalities) and large lesions assessed separately (*p *= 0.0055 for unenhanced MR and *p *= 0.0009 for Gd-BOPTA-enhanced MR).

No statistically significant difference in diagnostic efficacy between unenhanced MR and Gd-BOPTA-enhanced MR has been showed.

The sensitivity, specificity, PPV, NPV and accuracy of unenhanced versus gadolinium-enhanced MR and CT imaging in the characterization of liver hemangiomas are shown in table 4.

**Table 4:** Reported sensitivity, specificity, PVP, NPV and accuracy of multiphase sCT, Gd-BOPTA-enhanced MR and unenhanced MR studies in the characterization of hemangiomas divided into three groups according to size of diameter: small, large and all lesions.

	methods	sensitivity	specificity	PPV	NPV	accuracy
small lesions	multiphase sCT	0.66	0.85	0.65	0.86	0.79
	
	unenhanced MRI	1	1	1	1	1
	
	unenhanced MRI and Gd-BOPTA-enhanced MRI	1	1	1	1	1

large lesions	multiphase sCT	0.9	0.94	0.78	0.97	0.93
	
	unenhanced MRI	0.97	0.99	0.94	0.99	0.98
	
	unenhanced MRI and Gd-BOPTA-enhanced MRI	0.98	0.99	0.95	0.99	0.99

all size lesions	multiphase sCT	0.76	0.90	0.71	0.92	0.86
	
	unenhanced MRI	0.98	0.99	0.99	0.99	0.99
	
	unenhanced MRI and Gd-BOPTA-enhanced MRI	0.99	1	1	0.99	0.99

## Discussion

Differentiation between hemangiomas and malignant liver lesions is an important clinical problem both in patients with known malignancy as well as in cases of incidentally detected hepatic lesions.

The diagnosis of hemangiomas relies mainly on the following parameters: hyperechogenicity in ultrasound, typical enhancement pattern in CT or MR images (nodular peripheral in HAP with progressive fill-in in PVP and in EP) and high signal intensity in T2-weighted images [11-14].

In dynamic examination the enhancement pattern of hemangiomas does not differ in neither CT nor MR study.

Distinction between metastases and cavernous hemangiomas on the basis of different patterns of enhancement is well described, but to our knowledge, only few reports focus on the type of enhancement, diagnosed with hepatocyte-specific contrast-enhanced MRI [15].

In presented study the assessment of focal lesions in MR was performed with the use of moderately and heavily T2-weighted sequences and dynamic examination after Gd-BOPTA administration.

Ultrasound was the initial examination qualifying patients into this study as it allowed to distinguish a group of patients with ambiguous focal liver lesions, some of suspected malignant nature.

Patients presenting lesions of typical ultrasound appearance of hemangiomas and simple cysts were excluded from further studies as such lesions may be successfully monitored in grey-scale ultrasound examinations.

To assess foci in moderately T2-weighted images qualitative criteria proposed by Fenlon were used [16]. In moderately T2-weighted images, foci of markedly high signal intensity (so called bright lesions) were classified as hemangiomas (figure 2), assuming that they were homogenous. Lesions with a visible central necrosis were treated as heterogenous and changes in signal characteristics were referred to tissue elements on their periphery. Usefulness of T2-weigthed images for diagnosis of hemangiomas is already well known [7,17-23].

A high diagnostic efficacy of marked hyperintensity in moderately and heavily T2-weighted images as a feature allowing to diagnose hepatic hemangiomas was proven with indexes of diagnostic efficacy reaching 99% (table 4).

Similar observation was made by Ito et al. [17], who gained 100% efficacy in discriminating hemangiomas smaller that 3cm in diameter and McFarland et al., who reported that dual-echo heavily T2-weighted sequence (TE = 80, 160ms) allows to distinguish hemangiomas from malignant neoplasms with 100% sensitivity and 92% specificity [18] due to their highly hyperintense signal in T2-weighted images, described as the bulb-light sign [24-26].

Fenlon et al. compared qualitative and quantitative analysis of hepatic tumors using heavily T2-weighted SE technique and they noted that quantitative method with measurement of T2-relaxation times allowed significantly better differentiation between benign and malignant neoplasms with accuracy about 100% than that of the subjective visual assessment of focal liver lesions [16].

In our series a false-negative diagnosis concerned one case of hemangioma with areas of fibrosis and hyalinization, which mimicked a solid part of malignant necrotic neoplasm. One false-positive diagnosis of hemangioma was reported in case of cystic metastasis from ovarian cancer. We did not observe characteristic strong hyperintensity typical for hemangiomas in other cases of malignant tumors especially in necrotic or hypervascular metastases as noted in literature [27,28].

The next radiological feature that allows recognition of HH is the type of enhancement observed in successive phases of dynamic CT or MR examination after extracellular iodine or gadolinium based contrast medium administration. Ros et al. [29] distinguished three types of enhancement in liver hemangiomas according to their diameter:

- early homogenous enhancement seen in foci of less than 1.5 cm in diameter,

- nodular peripheral enhancement in HAP with a progressive centripetal fill-in pattern, to homogenous enhancement in the late phase (foci from 1.5 to 5cm in diameter),

- nodular peripheral enhancement in HAP with a hypointense center of the lesion in the late phase (foci larger than 5 cm in diameter).

Essentially in our study two types of enhancement patterns in HAP were stated: nodular peripheral and homogenous (table 1). At the same time correlation between type of enhancement and size of lesion was noted (table 2). In small hemangiomas, homogenous enhancement pattern was observed in HAP, PVP and EP phases in CT and MR studies. This finding was also reported by other authors [30-34] in MR examination and described as flash-filling pattern.

In hemangiomas greater than 2cm in diameter a nodular peripheral enhancement pattern in HAP with progressive slow centripetal fill-in during next phases has been observed. This type of enhancement has been described as specific for hemangiomas since 1986 [35-37].

The value of PPV and accuracy and sensitivity in differentiation of hemangiomas from other focal liver lesions based of enhancement pattern presented in multi-phase sCT study are 0.71, 0.86 and 0.76 and noticeably lower than all indexes of diagnostic efficacy obtained by using unenhanced-MRI (PPV-0.99). In the group of lesions smaller than 2cm, sensitivity of diagnostic methods based on contrast CT enhancement pattern is even lower, at the range of 0.66.

Presented study in accordance to the other authors [30,38] shows that nodular peripheral enhancement pattern allows discrimination of larger hemangiomas, while it has no application in differentiating small lesions.

In this study all hemangiomas presented weaker enhancement in hepatobiliary phase in comparison to the adjacent liver parenchyma, similarly to metastases and the majority of HCCs. This results from the characteristic of applied hepatocyte-specific contrast media - Gd-BOPTA. Gadobenate dimeglumine (Gd-BOPTA) is a gadolinium-based paramagnetic contrast with a vascular-interstitial distribution during first few minutes. The majority of this contrast agent is excreted by the kidneys in the urine, however, about 4% of the injected dose is taken up by the hepatocytes and eliminated via anionic transporter across the sinusoidal membrane into the bile [39].

Due to contrast agent presence within hepatic cells, it is possible to observe the enhancement of the liver parenchyma that persists for 1-3 hours after its administration and this period is called hepatobiliary phase.

A tumor that does not contain functioning hepatocytes in which hepatobiliary metabolism is active, is not able to uptake Gd-BOPTA.

Hemangiomas consist of multiple vascular channels limited by single layer of endothelial cells within a thin fibrous stroma and the enhancement in hepatobiliary phase is not observed, what makes hemangiomas impossible to differentiate from other hepatocyte-devoided liver tumors [25,40,41].

Main restrictions of this study are a small number of cases of hemangiomas confirmed in histological examination, lack of quantitative analysis of focal liver lesions signal intensity as described by other authors [42] and no incidence of giant hemangiomas (>8cm) in which secondary changes like hemorrhage or hyalinization are more frequent [43].

Our results indicate that qualitative analysis of signal intensity of hepatic lesions in moderately and heavily T2-weighted images is the most sensitive method of differentiation between liver hemangiomas of non-specific appearance in ultrasound from malignant tumors. Assessment of the enhancement pattern provides information about vascularity of the lesion although the type of enhancement pattern itself is not as important in characterization of hemangiomas as the intensity of the lesion in T2-weighted images.

Application of contrast-enhanced MR, especially with administration of liver-specific contrast agents in diagnosing liver hemangiomas is not advisable as it leads to elongation of examination time and unnecessary exposure to potential side effects of intravenous contrast agents.

## Conclusion

Gd-BOPTA-enhanced MR in comparison with unenhanced MRI does not improve diagnostic accuracy in discriminating hemangiomas that show non-specific appearance in ultrasound examination. Unenhanced MR as a method of choice should directly follow ultrasound examination in course of diagnostic algorithm in differentiation of hemangiomas from other liver tumors.

## Abbreviations

CT: computer tomography; EP: equilibrium phase; FNH: focal nodular hyperplasia; FSE: fast spin echo; HAP: hepatic arterial dominant phase; HBP: hepatobiliary phase; HH: hepatic hemangioma; MDCT: multidetector computer tomography; MR: magnetic resonance; MRI: magnetic resonance imaging; nonHH: other lesion than hemangioma; NPV: negative predictive value; PPV: positive predictive value; PVP: portal venous dominant phase; ROI: region of interest; sCT: spiral computed tomography; SE: spin echo; TE: echo time; TR: repetition time; US: ultrasound.

## Competing interests

The authors declare that they have no competing interests.

## Authors' contributions

ES has had substantial contribution to study design, data collection and interpretation, statistic analysis and manuscript preparation. TN has contributed to statistic analysis and manuscript preparation. EIS has contributed to data collection and interpretation, as well as manuscript preparation. JW and ADJ has contributed to study design and manuscript preparation. AS has contributed to data collection and interpretation. KM has contributed to manuscript preparation. MS has contributed to study design and has coordinated research team. All authors have read and approved the final manuscript.

## Pre-publication history

The pre-publication history for this paper can be accessed here:

http://www.biomedcentral.com/1471-230X/11/43/prepub
